# Paediatric Emergency Asthma Presentations: Temporal Trends and Representations in Rural Australia

**DOI:** 10.3390/healthcare11243113

**Published:** 2023-12-07

**Authors:** Daniel Terry, Blake Peck, Kate Kloot

**Affiliations:** 1 School of Nursing and Midwifery, University of Southern Queensland, Ipswich, QLD 4305, Australia; daniel.terry@unisq.edu.au; 2 Institute of Health and Wellbeing, Federation University, Ballarat, VIC 3350, Australia; b.peck@federation.edu.au; 3 School of Medicine, Deakin University, Warrnambool, VIC 3280, Australia

**Keywords:** asthma, paediatrics, emergency departments, healthcare utilisation, delivery of healthcare

## Abstract

Asthma is a key illness driving children to present to emergency departments, and although paediatric emergency asthma presentations have been examined, the temporal trends remain somewhat elusive. The aim is to highlight, describe, and model the temporal trends of emergency paediatric asthma presentations, using comprehensive hospital emergency presentation data. A retrospective cross-sectional study examined de-identified paediatric (0 to 14 years) emergency asthma presentation data over a three-year period. Data were obtained from nine healthcare facilities in Victoria, Australia. Episode-level data were collected through RAHDaR, a comprehensive emergency data register which includes missing data (35.0%) among rural health facilities not currently captured elsewhere. Monthly presentation rates demonstrate a significant difference in presentations between fall/autumn and spring, and males had higher presentation rates in February and June–August. Emergency presentations were more likely to occur Sunday–Tuesday, peaking in the time periods of 8–9 a.m., 11 a.m.–12 p.m., and 8–9 p.m. Significant differences were noted between all age groups. Examining previously unavailable rural data has highlighted patterns among emergency asthma presentations for children 0–14 years of age. Knowledge of these by season, month, and day of the week, in combination with time of day, offers scope for more focused workforce education and planning, and nuanced referral pathways, particularly in resource-limited settings.

## 1. Introduction

Asthma remains a pervasive chronic illness among children presenting as airway inflammation with reversible bronchial narrowing and obstruction [1,2]. Although mostly preventable, seeking emergency care is sometimes appropriate and vital [1,2]. More than 10.0% of the Australian population have been diagnosed with asthma, with similar rates observed among children [3,4]. Notwithstanding the implementation of national guidelines, education campaigns, and individualised self-management plans, asthma-related illness continues to impact healthcare [1,3].

Asthma is a key illness driving children to present to emergency departments in Australia. Although paediatric emergency asthma presentations (EAPs), which include children aged 0–14 years of age, with a principal diagnosis being asthma or asthma-related, have been examined, the temporal trends remain somewhat elusive [5]. This is exacerbated in areas of the country where state-wide hospital data are not centrally collated and up to a third of EAP data, specifically in rural areas, are not readily accessible [5,6]. These health services are often resource-limited, smaller rural hospitals, with reduced access to staff and auxiliary services outside of business hours [7]. As facility capabilities can vary greatly in rural areas, particularly in terms of availability of specialist paediatric staffing [7], understanding presentation trends can aid in pre-planning, including the timing of education, resourcing considerations, and/or referral pathway development.

Within this context, the aim of this study is to highlight, describe, and model, using a comprehensive hospital emergency presentation data register, the temporal trends of emergency paediatric asthma presentations to Southwest Victorian hospitals, a known asthma hospitalisation area [2].

## 2. Materials and Methods

A retrospective cross-sectional study was conducted to examine all de-identified paediatric EAP data between 1 February 2017 and 31 January 2020 among children aged 0 to 14 years as previously described [5]. This current dataset differs slightly from that previously published, as an additional 12 months of presentation data have been captured, while non-resident presentations are also included in order to fully capture resourcing implications at the facility level. It must be noted there were no known unusual environmental factors (thunderstorm asthma, etc.) that may have created clear spikes in asthma EAP incidents throughout the four-year study period.

### 2.1. Data Collection

Data were obtained from nine healthcare facilities across six Local Government Areas (LGA) in South West Victoria, an area within the state of Victoria identified as a “hotspot” for asthma exacerbations and hospitalization [2,5]. Collectively, the LGAs encompass a population of 128,058, which includes 22,627 (17.6%) children aged 0–14 years of age living across 26,322 square kilometres (16,355 square miles) [8]. Episode-level data were collected through the Rural Acute Hospital Data Register (RAHDaR) [6], which accurately collates data from participating healthcare facilities who provide emergency or urgent care across the region’s six LGAs [5,6].

RAHDaR data are based on the Victorian Emergency Minimum Dataset (VEMD) (version 22 for 2017–2018) [6] currently mandated to be reported to the state government regarding presentations that occur at designated emergency departments (ED) in larger or well-resourced health services. However, RAHDaR also includes emergency presentation data from Urgent Care Centres (UCC), providing urgent or critical access services at less resourced and more rural health facilities. Specifically, UCCs provide first-line emergency care and have the capacity to perform emergency resuscitation and stabilisation of patients and, where clinically appropriate, prepare patients for transfer to a higher level of care. However, care may be limited [5]. In most cases, UCCs do not have a pulmonologist or paediatrician onsite, but are staffed by highly trained nursing staff and on-call general practitioners (family physicians) [5].

RAHDaR data, designated as non-Victorian Emergency Minimum Dataset (non-VEMD), is not routinely captured or reported to the state government, and represents at least 35.0% of all state-wide emergency data that are not used to inform emergency resourcing requirements [2]. 

Within this study, two of the nine health services had EDs and were designated as VEMD health services, while the remaining seven services were UCCs and designated non-VEMD health services. It is noted a single emergency event may result in several emergency presentations, specifically, when an individual is transferred between institutions. In the event a transfer occurred, all presentations were included to ensure all health service usage data was accounted. 

Data from RAHDaR were extracted with the principal diagnosis being asthma-related (ICD-10-AM: J45.0, J45.1, J45.8, J45.9 and J46.0) [9]. Data fields included gender; age; LGA; postcode; date and time of arrival and departure; and departure status. As guided by the work of Terry et al. [2], additional LGA level data were obtained, including 2017–2020 population data to ensure the demographic evolution of the population were calculated over the three-year period [8].

### 2.2. Data Analysis

Population figures for each LGA were used to calculate EAP incidence rates across the study region. EAP events were divided by the corresponding population estimates for each year according to gender, age group, and health service type (ED or UCC) to obtain age-standardised EAP incidence rates [10]. Lastly, data were also triangulated by gender, age, and postcode to estimate multiple presentations of individuals. 

Emergency asthma presentation data were analysed across the various facilities using Statistical Package for the Social Sciences (SPSS, Version 25.0). ANOVA and *t*-tests were used to examine the association between EAP, month, season, age group, and gender. In addition, Chi-square (χ^2^) tests were used to determine if asthma presentations differed between gender and age groups according to the day, month, and time of day the EAP occurred. Significance was two-tailed *p* ≤ 0.05.

### 2.3. Ethical Approval

The study was conducted in accordance with the Declaration of Helsinki and has ethical approval from South West Healthcare (SWH-2019-167567), Federation University Australia (FUHREC E19-005), and Deakin University (DUHREC 2019-135).

## 3. Results

Over the three-year period, 1205 EAPs occurred among 956 children. Asthma-related presentations represented 3.8% of the total paediatric emergency presentations across the nine health services, the second-largest proportion of presentations following injuries. The largest proportion of asthma presentations occurred in the three-month period to April 2017 followed by the three-month period to October 2017 (Table 1).

The 1205 asthma presentations included both residents (90.4%) and visitors to the region (9.6%). The highest incidence occurred among males (19.03 presentations per 1000 children per year) and children aged 0–4 years (25.82 presentations per 1000 children per year) (Table 2). Lastly, 249 (20.6%) of all presentations were estimated a second or subsequent emergency presentation of children; 91 (36.4%) of these occurring within a 30-day period. Two children attended an emergency or UCC more than 11 times over the period.

### Timing of Visit

Monthly presentation rates were plotted, where peaks in presentation rates occurred annually in May (end of fall/autumn) and November (end of spring), while troughs in presentations occurred in September (early spring), December, and January (summer) (Table 3). Significant differences in presentation rates occurred between the various seasons, F(3, 1204) = 37.099, *p* < 0.001. Summer (December to February) had lower presentation rates compared with all other seasons, while fall/autumn (March to May) had much higher presentation rates compared to spring (September to November). Seasonal presentations between each age group were examined, and children aged 0–4 had higher presentation rates in fall/autumn (March to May) compared to children aged 10–14 years of age, F(2, 328) = 3.175, *p* = 0.03.

Monthly presentations among genders demonstrated similar peaks and troughs. However, males had a higher proportion of presentations in February, June, July, and August compared to females with a significant difference detected, t(322) = 23.227, *p* < 0.001. Males were significantly more likely to have higher presentation rates in winter (June–August) (Table 4).

When examining attendance by the day of the week, emergency presentation rates were much higher and more likely to occur on Sundays, Mondays, and Tuesdays (Table 5). When plotted against gender, significant differences were observed for each day of the week. A higher rate of presentations among males was seen each day, except on Thursdays and Sundays, t(1204) = 50.215, *p* < 0.001. However, there were no significant differences in presentation rates between each of the various age groups according to the day of the week, F(2, 1204) = 0.399, *p* = 0.76.

When examining the time of day each presentation occurred, there was a propensity for emergency presentations to peak in the time periods of 8–9 a.m., 11 a.m.–12 p.m., and again at 8–9 p.m. (Table 6). No differences were demonstrated between males and females, t(1204) = 0.284, *p* = 0.72. However, significant differences according to age group and presentation hours were detected throughout most of a 24 h period, F(2, 1204) = 334.541, *p* ≤ 0.001. Higher rates occurred among the 0–4-year-old age group than the 5–9 and 10–14-year-old age groups, with presentation rates much higher between 8 a.m. and 12 p.m., with a peak at 4 p.m., and increasing between 6 and 8 pm. Conversely, the 5–9- and 10–14-year-old age groups were more likely to present to emergency between 9 p.m.–10 p.m. (Table 5).

## 4. Discussion

When examining a complete dataset of paediatric asthma presentations to an Emergency Department or Urgent Care Centre, key characteristics have been identified that may assist to inform resourcing requirements, particularly for those health services where data are not routinely centrally collated. Consistently with existing national [11] and international [12] research, both age and gender were associated with differences in EAPs among children, a steady trend since 2001 [4]. These differences have been linked to the effects of various asthma phenotypes, with viral infections influencing young and immunologically susceptible children during winter, while incidence amongst school age children fluctuates in accordance with the school term [11,12,13]. Both explanations find consistency with our data. It is noted that virally linked asthma has a tendency to occur among much younger children (0–6 years of age), which may account for children aged 0–4 having significantly higher presentation rates in fall/autumn months compared to children aged 10–14 years of age [13]. Further, the higher presentation rates among males throughout winter months may also be related to a relatively smaller airway size compared to females under 10 years of age [13]. As such, males have a propensity to have increased reactivity associated with environmental factors such as the cold weather, seasonal allergens, and triggers such as smoke, moulds, and dust.

With regard to allergic asthma phenotypes, the data are consistent with other Australian studies demonstrating an annual cycle of increased presentations seen in the month of May (autumn/fall) [11]. Interestingly, the study by Simunovic et al. [11], conducted in Queensland, highlights a second peak in August which coincides with elevations in aeroallergens in the region. Outside the winter period, aeroallergens may partly contribute to the observed results, albeit only exerting a small and inconsistent increase on emergency presentations for asthma [14]. In this study the second peak is seen in early November. South West Victoria is predominantly rural farmland, and, with the peak of the grass pollen season in Victoria being early November [15], it is reasonable to assume some influence on EAPs. Longitudinal knowledge of asthma and its characteristics in Victoria, particularly in rural regional areas remains absent [2], despite studies that target this region [2]. Identification of pollen-related asthma presentation trends may assist the timing of appropriate staff education to enable upskilling in less common, but potentially fatal, presentations [16].

The variation in hour and day patterns within the data has been explained elsewhere as general fluctuations in Emergency Department utilization [17]. Specifically, the timing of the visit on a Sunday, Monday, or Tuesday may represent a complex interaction of factors centred around a tension between parental, particularly maternal, employment commitments and childcare [18]; the most convenient time for parent to transport their children to the Emergency Department or Urgent Care Centre [17]; and an underestimation of their child’s condition by the parent [18]. Alternatively, it may be when least convenient to visit an alternative healthcare provider, such as a family physician or general practitioner [19]. Current patterns warrant further investigation, particularly as weekend presentations may have a larger impact on resource-limited facilities.

This is the first Australian study to characterise EAPs to the level of days and time of the week, while Sunday and Monday have routinely been identified as days of high frequency for paediatric EAPs in New Zealand [20] and Canada [17]. This phenomenon observed in the Canadian adult population was purported to be associated with limited access to family physicians or clinics on weekend. However, it has also been suggested that weekends are also associated with undertaking greater numbers of outdoor or physical activities [21], all of which may impact the higher rates of Sunday and Monday emergency presentations observed [21]. However, it may be argued that attendance at school and daycare during the previous week, where virally induced exacerbations or more time spent outside for allergen-induced asthma, may lead to a greater propensity for weekend EAPs. Although outside the scope of this study, it highlights the need to undertake additional research.

Nevertheless, within this study, it was identified males had higher presentation rates than females throughout the week, except Thursdays and Sundays, where rates were similar. These similar rates are comparable to other studies demonstrating overall presentations are their lowest on Thursdays [17], while further suggesting limited primary healthcare access on weekends may explain similarities on Sundays [21]. Knowledge regarding the timing of the asthma presentations among children remains currently limited within the literature.

In addition, Rosychuk et al. [17], identified 8 a.m. to 11 a.m. and 7 p.m. to 11 p.m. as the two peaks for paediatric presentations in Canada. While reflective of this study, the length of the peaks in this data are shorter; however, they may be accounted for by specific geography, population density, or healthcare nuances that differ between these comparable countries [22]. The examination of presentation timing by age group, a finding not noted in other studies, may offer a more nuanced insight to inform resource planning to meet service demand, particularly in smaller rural health services with fewer resources.

Evidence suggests 30 days or less between initial hospital presentation and readmission for exacerbations of asthma is potentially associated with higher acuity, less well-controlled asthma, or challenges associated with asthma management planning or adherence [12]. Within the limitations of the methodology, our data deduced several children (21.1%) presented to the ED/UCC a second or subsequent time within the three-year study period. Of this cohort, more than one third (36.4%) were estimated to have two or more exacerbations within 30 days of their initial visit. This suggests these paediatric EAPs may present with less well controlled and/or potentially higher acuity asthma, and thus more resource intensive, a phenomenon which is not well addressed within the existing literature. Health professionals who do not specialise in paediatric care, such as those in rural UCCs, are more likely to have lower confidence managing children [23], therefore identifying the need for additional education and/or staffing based on facility-specific trends that may improve morale and staff retention [24].

Paediatric asthma represents a significant challenge to EDs/UCCs, particularly in rural health services where there is limited staffing and access to on duty general practitioners, pulmonologists, or paediatricians. Despite improvements in understanding and management of asthma interventions over the past few decades, further reducing its exacerbation among children remains somewhat elusive. However, using data more accurately to inform resourcing (specialised nursing staff at peak times, access to telemedicine, or medical staff), and timely education (continued professional development, specialised rural health training, asthma specific education) further supports health services to meet peak demands, particularly in rural and resource-limited settings. Such insights may guide smaller rural health services to identify when further expertise may be needed, if additional training is required prior to peak seasons, or when telehealth is necessary to meet peak demand. Such understanding also provides insight into improved accessibility; referral pathway development and utilisation; and follow up.

### 4.1. Limitations

Study limitations include the use of retrospective emergency presentation data and the lack of arrival mode and acuity information. Although the triangulation of data supported the understanding of emergency re-presentations, it may not account for twins or the rare possibility that two children from the same town with the same gender and date of birth may have presented over this time. In addition, the 1205 presentations across the nine facilities over the three years represented an average of 44.6 presentations per facility per year (range 8.0 to 224.3) and the paediatric EAPs may be considered too rare to enable meaningful implications. An additional limitation pertains to the criteria for diagnoses of asthma, where a diagnosis of asthma is very hard to establish and confirm among 0–4-year-olds. Although based on ICD-10-AM, these diagnoses may be difficult in smaller rural healthcare settings where paediatric specialist availability is limited. As such, this may lead to misdiagnosis and account for over- or underestimation of EAPs within the dataset. A major study strength is the use of the previously missing 35% of emergency presentation data from the smaller rural hospitals, which provides nuanced insights to facilitate greater targeted programs and research to support children and families.

### 4.2. Clinical Implications

The clinical implications pertain to the ability to enhance pre-planning in resource-limited settings, specifically human resourcing, skill sets, and clinical care approaches, particularly among smaller, rural, and less resourced health services. There are several known seasonal predictors that help to understand broader peak demand. However, the more nuanced peak demands such as the day of the week and time of day further inform how or in what capacity increased emergency asthma demand may be better addressed, particularly when resources at these times may already be limited.

## 5. Conclusions

Paediatric asthma represents a significant burden on emergency departments globally. Despite improvements in both our understanding and management of asthma interventions over the past few decades, further reducing its exacerbation among children who may require emergency care remains somewhat elusive. However, the use of data to predict resourcing requirements more accurately, particularly in resource-limited settings, better supports health services to upskill staff and meet peak demands, while also providing insight into improved accessibility, referral pathways, and follow-up. This study examining previously unavailable data has highlighted patterns among asthma emergency presentations for children 0–14 years of age. Variations in length of stay as well as the rates of repeat presentation amongst this cohort offers an opportunity for more focused healthcare approaches and workforce planning, and more nuanced referral pathways, along with future research. A demonstration that season, month, and day of the week, in combination with the time of day, offers some scope for future investigation to better understand the nuances of these variables and their influence on presentation rates. Each visit to an emergency or urgent care setting is an opportunity to provide a child and their family with an intervention that may prevent future presentations. Data such as this offer an opportunity for healthcare providers to tailor both human and physical resources to better manage the peak periods of presentations, and in so doing, provide further and ongoing support to families and children seeking to independently manage asthma in the future.

## Figures and Tables

**Table 1:** Proportion of paediatric asthma presentations compared to other paediatric presentations over the study period.

Time Period (Three Months to Date)	Asthma Presentations (Percentage %)	Non-Asthma Presentations (Percentage %)
1 January 2017	3.6%	96.4%
1 April 2017	5.1%	94.9%
1 July 2017	3.3%	96.7%
1 October 2017	4.9%	95.1%
1 January 2018	3.0%	97.0%
1 April 2018	4.5%	95.5%
1 July 2018	3.5%	96.5%
1 October 2018	3.6%	96.4%
1 January 2019	3.7%	96.3%
1 April 2019	3.2%	96.8%
1 July 2019	3.4%	96.6%
1 October 2019	3.4%	96.6%
1 January 2020	3.3%	96.7%

**Table 2:** Demographic data of emergency asthma presentations.

Factor	Number	Percentage	Emergency Asthma Presentation Incidence Rate †
Year (*n* = 1205)			
2017/2018 (February–January)	430	35.7%	6.33
2018/2019 (February–January)	377	31.3%	5.56
2019/2020 (February–January)	398	33.0%	5.86
Gender (*n* = 1205)			
Female	534	44.3%	16.37
Male	671	55.7%	19.03
Age group (*n* = 1205)			
0–4 years	546	45.3%	25.82
5–9 years	420	34.9%	18.08
10–14 years	239	19.8%	10.16
Location presented (*n* = 1205)			
UCC	433	35.9%	6.37
Emergency	772	64.1%	11.37
Residence (*n* = 1205)			
Southwest Victoria	1089	90.4%	16.04
Other areas of state	98	8.3%	-
Interstate	16	1.4%	-
Overseas	1	0.1%	-
Unknown	1	0.1%	-
Number of presentations per child (*n* = 1205)			
One	956	79.4%	-
Two	128	10.6%	-
Three	58	4.8%	-
Four	23	1.9%	-
Five	19	1.6%	-
Six or more	21	1.7%	-
Repeat presentations within 30-day period (*n* = 250)	91	36.4%	-

† Presentations per 1000 children per year.

**Table 3:** Rate of presentations according to month and age group.

Month	0–4 Years of Age			5–9 Years of Age			10–14 Years of Age		
	Incident Rate †	95% CI		Incident Rate †	95% CI		Incident Rate †	95% CI	
January	2.554	2.517	2.590	1.809	1.784	1.833	2.679	2.643	2.715
February	6.526	6.433	6.618	2.455	2.421	2.488	4.338	4.280	4.397
March	5.675	5.594	5.755	3.488	3.441	3.535	3.317	3.273	3.362
April	7.093	6.993	7.194	2.842	2.804	2.881	3.445	3.399	3.491
May	8.654	8.531	8.777	3.101	3.059	3.143	6.762	6.671	6.853
June	7.235	7.132	7.338	2.455	2.421	2.488	5.869	5.790	5.948
July	6.809	6.713	6.906	1.679	1.657	1.702	4.211	4.154	4.267
August	7.944	7.832	8.057	2.713	2.676	2.750	4.338	4.280	4.397
September	4.540	4.475	4.604	2.325	2.294	2.357	2.935	2.895	2.974
October	5.533	5.454	5.611	2.196	2.166	2.226	4.593	4.531	4.655
November	8.228	8.111	8.345	3.876	3.823	3.928	6.507	6.420	6.595
December	6.526	6.433	6.618	1.938	1.912	1.964	4.721	4.657	4.784

† Presentations per 1000 children per year.

**Table 4:** Rate of presentations according to month and gender.

Month	Male			Female			Total		
	Incident Rate †	95% CI		Incident Rate †	95% CI		Incident Rate †	95% CI	
January	0.794	0.771	0.817	0.797	0.773	0.822	0.796	0.772	0.819
February	1.701	1.679	1.724	1.196	1.171	1.220	1.458	1.435	1.482
March	1.361	1.338	1.384	1.380	1.355	1.404	1.370	1.346	1.394
April	1.418	1.395	1.441	1.502	1.478	1.527	1.458	1.435	1.482
May	1.957	1.934	1.979	2.146	2.122	2.171	2.048	2.024	2.071
June	1.900	1.877	1.923	1.502	1.478	1.527	1.709	1.685	1.732
July	1.758	1.735	1.781	0.981	0.957	1.006	1.385	1.361	1.408
August	1.957	1.197	1.979	1.318	1.294	1.343	1.650	1.243	1.674
September	1.021	0.998	1.044	1.104	1.079	1.128	1.061	1.037	1.084
October	1.531	1.027	1.554	1.134	1.110	1.159	1.341	1.067	1.364
November	2.098	1.792	2.121	1.962	1.938	1.987	2.033	1.862	2.057
December	1.531	1.225	1.554	1.349	1.325	1.374	1.444	1.273	1.467

† Presentations per 1000 children per year.

**Table 5:** Rate of presentations according to the day of the week and gender.

Day	Male			Female			Total		
	Incident Rate †	95% CI		Incident Rate †	95% CI		Incident Rate †	95% CI	
Monday	3.034	2.643	3.425	2.391	2.063	2.720	2.725	2.364	3.086
Tuesday	3.204	2.813	3.596	2.606	2.278	2.934	2.917	2.556	3.278
Wednesday	2.892	2.501	3.284	2.238	1.910	2.567	2.578	2.217	2.939
Thursday	2.155	1.764	2.546	2.177	1.848	2.505	2.166	1.804	2.527
Friday	2.354	1.962	2.745	1.962	1.634	2.291	2.166	1.804	2.527
Saturday	2.467	2.076	2.858	2.085	1.756	2.413	2.283	1.922	2.644
Sunday	2.921	2.529	3.312	2.913	2.584	3.241	2.917	2.556	3.278

† Presentations per 1000 children per year.

**Table 6:** Rate of presentations according to the time of the day and age group.

Hour	0–4 Years		5–9 Years		10–14 Years		Total	
	Incident Rate *	95% CI	Incident Rate *	95% CI	Incident Rate *	95% CI	Incident Rate *	95% CI
1	0.426	0.087–0.765	0.388	0.387–0.726	0.255	0.117–0.534	0.354	0.249–0.456
2	0.520	0.312–0.633	0.431	0.429–0.591	0.212	0.174–0.687	0.383	0.228–0.480
3	0.473	2.778–0.663	0.258	0.258–0.468	0.213	0.027–0.393	0.309	0.198–0.420
4	0.709	0.576–0.840	0.560	0.558–0.702	0.340	0.159–0.519	0.530	0.447–0.615
5	0.284	0.075–0.492	0.517	0.516–0.663	0.340	0.159–0.519	0.397	0.285–0.483
6	0.378	0.198–0.558	0.474	0.471–0.627	0.085	0.012–0.156	0.309	1.980–0.420
7	0.757	0.630–0.882	0.344	0.342–0.525	0.085	0.012–0.156	0.383	0.285–0.483
8	1.229	1.106–1.314	0.818	0.816–0.936	0.510	0.363–0.657	0.839	0.776–0.964
9	1.277	1.179–1.377	0.473	0.413–0.563	0.298	0.105–0.489	0.662	0.603–0.733
10	1.561	1.443–1.617	0.775	0.774–0.894	0.383	0.213–0.555	0.884	0.819–0.951
11	1.939	1.860–2.016	0.775	0.774–0.894	0.425	0.264–0.588	1.016	0.957–1.077
12	1.229	1.089–1.289	0.775	0.774–0.918	0.510	0.315–0.621	0.825	0.711–0.889
13	1.182	1.080–1.284	0.732	0.732–0.855	0.383	0.213–0.555	0.751	0.678–0.822
14	0.851	0.732–0.972	0.689	0.645–0.738	0.595	0.459–0.729	0.707	0.648–0.768
15	0.945	0.822–1.103	0.775	0.774–0.918	0.5553	0.333–0.687	0.751	0.678–0.822
16	1.561	1.455–1.665	0.775	0.774–0.870	0.383	0.213–0.555	0.884	0.819–0.951
17	1.277	1.356–1.554	1.206	1.203–1.326	0.298	0.105–0.489	0.913	0.846–0.978
18	1.561	1.473–1.647	1.077	1.074–1.179	0.510	0.363–0.657	1.046	0.972–1.142
19	1.655	1.569–1.743	1.292	1.203–1.382	0.680	0.553–0.778	1.193	1.092–1.226
20	2.222	1.983–2.289	1.334	1.254–1.419	0.595	0.459–0.729	1.355	1.197–1.412
21	0.993	0.880–1.084	1.292	1.29–1.386	1.106	1.098–1.206	1.134	1.044–1.194
22	1.135	1.029–1.239	1.077	1.074–1.179	0.553	0.453–0.632	0.913	0.839–0.951
23	0.804	0.681–0.927	0.732	0.732–0.855	0.425	0.264–0.588	0.648	0.570–0.726
24	0.851	0.732–0.972	0.431	0.429–0.594	0.425	0.264–0.588	0.560	0.477–0.645

* Presentations per 1000 children per year.

## Data Availability

The data that support this study cannot be publicly shared due to ethical or privacy reasons and may be shared upon reasonable request to the corresponding author if appropriate.
